# Down Syndrome Is a Metabolic Disease: Altered Insulin Signaling Mediates Peripheral and Brain Dysfunctions

**DOI:** 10.3389/fnins.2020.00670

**Published:** 2020-07-08

**Authors:** Mara Dierssen, Marta Fructuoso, María Martínez de Lagrán, Marzia Perluigi, Eugenio Barone

**Affiliations:** ^1^Centre for Genomic Regulation (CRG), Barcelona Institute of Science and Technology, Barcelona, Spain; ^2^Universitat Pompeu Fabra, Barcelona, Spain; ^3^Human Pharmacology and Clinical Neurosciences Research Group, Neurosciences Research Program, Hospital Del Mar Medical Research Institute (IMIM), Barcelona, Spain; ^4^Centro de Investigación Biomédica en Red de Enfermedades Raras (CIBERER), Madrid, Spain; ^5^Department of Biochemical Sciences “A. Rossi-Fanelli”, Sapienza University of Rome, Rome, Italy

**Keywords:** Down syndrome, metabolism, insulin, brain insulin resistance, meatbolic disroders

## Abstract

Down syndrome (DS) is the most frequent chromosomal abnormality that causes intellectual disability, resulting from the presence of an extra complete or segment of chromosome 21 (HSA21). In addition, trisomy of HSA21 contributes to altered energy metabolism that appears to be a strong determinant in the development of pathological phenotypes associated with DS. Alterations include, among others, mitochondrial defects, increased oxidative stress levels, impaired glucose, and lipid metabolism, finally resulting in reduced energy production and cellular dysfunctions. These molecular defects seem to account for a high incidence of metabolic disorders, i.e., diabetes and/or obesity, as well as a higher risk of developing Alzheimer’s disease (AD) in DS. A dysregulation of the insulin signaling with reduced downstream pathways represents a common pathophysiological aspect in the development of both peripheral and central alterations leading to diabetes/obesity and AD. This is further strengthened by evidence showing that the molecular mechanisms responsible for such alterations appear to be similar between peripheral organs and brain. Considering that DS subjects are at high risk to develop either peripheral or brain metabolic defects, this review will discuss current knowledge about the link between trisomy of HSA21 and defects of insulin and insulin-related pathways in DS. Drawing the molecular signature underlying these processes in DS is a key challenge to identify novel drug targets and set up new prevention strategies aimed to reduce the impact of metabolic disorders and cognitive decline.

## Introduction

Down syndrome (DS) is the most frequent chromosomal abnormality that causes intellectual disability, resulting from the presence of an extra complete or segment of chromosome 21 (HSA21). Trisomy of HSA21 is associated not only with intellectual disability but also with several morphological and physiological features. Several regions of the HSA21 contribute to DS clinical manifestations, as suggested by a number of studies performed in human bearing partial duplication (Lejeune et al., 1959; Patterson, 2009). However, past studies mainly focused on the “Down syndrome critical region (DSCR)” as one of the main candidate regions considered to be responsible for the majority of DS features as a direct consequence of down- or up-regulation of its target (Rachidi and Lopes, 2007). The DSCR region includes important candidate genes, such as the dual-specificity tyrosine (Y)-phosphorylation regulated kinase 1 A (DYRK1A), involved in brain development and learning and memory (Altafaj et al., 2001; Park et al., 2009); the regulator of calcineurin 1 (RCAN1), which plays a role in cell growth and immune responses but has also a role in cognition (Dierssen et al., 2011; Mendez-Barbero et al., 2013); the cystathionine beta-synthase (CBS), an enzyme involved in the homocysteine/folate/transulfuration pathways (Iacobazzi et al., 2014); and superoxide dismutase 1 (SOD1), which helps redox homeostasis (Valko et al., 2016). All these genes have been further characterized by the phenotypic and molecular analysis of transgenic mice expressing every single gene separately, and collected data demonstrate many common pathological features with the whole HSA21 genotype (Roper and Reeves, 2006). Among other HSA21 candidates, the triplication of some microRNAs (miRNAs), in particular miR-155 (Mashima, 2015), may further contribute to modulate target genes leading to changes of neurochemical metabolites, mitochondrial deficits, and other pathological conditions observed in DS individuals (Quinones-Lombrana and Blanco, 2015). Further, experimental evidence obtained in trisomic and monosomic mouse models allowed identification of regions outside the DSCR that are responsive to dosage that may contribute to defects in behavior and cognition and other DS pathological features (Vacano et al., 2012; Gupta et al., 2016).

The most characteristic trait of DS individuals is disturbance of brain development associated with intellectual disability in the presence of craniofacial abnormalities (Richtsmeier et al., 2002). Further, the brains from DS subjects have structural and functional deficits associated with defects in morphogenesis, leading to significant reduced brain volume (Haydar and Reeves, 2012).

Most DS individuals, by their 40s, develop a type of dementia that shares many commonalities with Alzheimer’s disease (AD), such as the deposition of senile plaques and neurofibrillary tangles (Head et al., 2016). After the age of 50, the risk of developing AD-like dementia rises in DS patients up to 70%. The high incidence of the symptoms characteristic of AD in people with DS is thought to be due to triplication of genes already demonstrated to be involved in AD pathology, including amyloid precursor protein (*APP*), beta secretase 2 (*BACE2*), *SOD1*, and S100 calcium-binding protein B (*S100B*), among others (Wiseman et al., 2015). The overexpression of *APP*, an integral membrane protein that is concentrated in neuronal synapses, and the consequent overproduction of amyloid beta (Aβ)-peptide is considered one of the major toxic players in the early onset of AD neuropathology in DS population (Hartley et al., 2015; Wiseman et al., 2015). It is well-known that Aβ overload is involved in the increased production of reactive oxygen species (ROS), elevated oxidative stress, and causes excitotoxicity, disturbance of cellular respiration and of synaptic functions. Accumulation of Aβ in DS brain can be observed as early as 8–12 years of age and increases during the patient’s lifespan (Head et al., 2016). Moreover, dementia features appear in DS adults in their 50s, suggesting a prodromal phase where subtle clinical signs are undetectable, although in young adults, worse performance in semantic verbal fluency test and poorer communication skills are associated with higher plasma Aβ42 concentrations and impaired communication skills (ABAS–II) (Hoyo et al., 2015). The exact mechanisms through which trisomy 21 causes early onset of AD-like neuropathology and cognitive decline, however, need further studies (Head et al., 2016).

## Down Syndrome as a Metabolic Disease

Dr. Jerome Lejeune was the first to hypothesize that DS could be considered a “metabolic disease.” This is highlighted by Caracausi et al. (2018): “in the conference talk “*Vingt Ans Après*,” he explained how the one carbon cycle could be involved in the pathogenesis of intellectual disability in subjects who do not have a gross anatomic defect of the brain, and he asserted: “the goal is to figure out where a link between mental deficiency and trisomy 21 should be sought.”

To confirm the “Lejeune hypothesis,” a number of alterations on metabolite concentrations have been observed in the blood of individuals with DS compared with age-matched control subjects, in particular alteration of a number of amino acids (Caracausi et al., 2018). Specifically, in plasma from DS subjects it has been reported to have: (i) increased levels of phenylalanine and tyrosine; (ii) reduced levels of histidine, lysine, tyrosine, phenylalanine, leucine, isoleucine, and tryptophan; (iii) higher levels of leucine, isoleucine, cysteine, and phenylalanine at an age vulnerable to Alzheimer’s changes; (iv) decreased concentration of serine at any age; and (v) increased lysine concentration in patients above 10 years old, possibly associated to accelerated aging, as reviewed in Caracausi et al. (2018). A recent study also evidenced changes in the levels of metabolites involved in the methylation cycle, including cysteine, cystathionine, choline, and dimethylglycine. Mass spectrometry analysis reported a significant increase of the concentrations of these amino acids in DS plasma (Obeid et al., 2012) as well as the levels of S-adenosylhomocysteine and S-adenosylmethionine (Obeid et al., 2012), though a previous study showed to be decreased (Pogribna et al., 2001).

The levels of these metabolites seem to be strongly associated with triplication of CBS (Skovierova et al., 2016). As recently reported, overexpression of CBS is responsible for reduced homocysteine levels together with reduced mitochondrial function, as indicated by accumulation of Krebs cycle intermediaries in DS human dermal fibroblasts (Panagaki et al., 2019). In particular, Szabo et al. showed that increased production of H_2_S is responsible for mitochondrial deficits, in particular by inhibiting complex IV (Panagaki et al., 2019). These results confirmed that both cytosolic and mitochondrial CBS protein levels, as well as H_2_S cellular levels, are markedly elevated in DS fibroblasts. Extracellular flux analysis of DS cells showed a significant impairment of mitochondrial complexes activities and oxygen consumption, and of ATP generation, which also affected proliferation rate. The specific activity of Complex IV was found to be significantly inhibited in DS cells. Interestingly, the inhibition of CBS by aminooxyacetate (AOAA), which interferes with the pyridoxal phosphate in the catalytic site, restored Complex IV activity and ameliorated mitochondrial electron transport and cell proliferation. Similar effects were also obtained by CBS normalization by siRNA (Panagaki et al., 2019). Moreover, increased urinary thiosulfate [a stable degradation product of hydrogen sulfide (H_2_S)] and circulating sulfhemoglobin (addition of H_2_S to hemoglobin) levels have already been detected in subjects with DS (Szabo, 2020).

Formimidoyltransferase cyclodeaminase (*FTCD*) is another gene located on the long arm of HSA21, which encodes an enzyme that participates in histidine and folate metabolism, both essential for purine, pyrimidines, and amino acids biosynthesis. In addition, aberrant metabolism of adenosine, homocysteine, and folate was also observed in DS (Patterson, 2009; Gross et al., 2019).

Among key regulatory metabolic enzymes, HSA21 also encodes for phosphofructokinase (PFK), a regulatory enzyme in glycolysis as it catalyzes the phosphorylation of fructose-6-phospate to fructose-1, 6-bisphosphate (Uyeda, 1979). Interestingly, transgenic mice that overexpress PFK liver type (Tg- PFKL) showed alteration in glucose metabolism as indexed by increased metabolic flux in brain and reduced clearance from blood (Peled-Kamar et al., 1998). The increased glucose utilization in the brain of Tg-PFKL mice is similar to the observed faster glucose metabolism in young DS adult brain that likely contributes to cognitive disabilities. Further, a previous report showed that PFK specific activity is twofold higher in the brains of embryonic Tg-PFKL mice (Elson et al., 1994), suggesting that aberrant glucose metabolism is already pronounced in developmental period and that this early dysmetabolism may contribute to learning disabilities. These age-dependent changes of gene expression further complicate the “gene-dosage effects” hypothesis of trisomy 21, contributing to the multifaceted aberrant metabolism observed at different ages (Pelleri et al., 2018).

Furthermore, DS subjects are characterized by an impaired lipid metabolism, although this aspect has been less investigated. Published studies report that DS children show higher levels of circulating cholesterol, low-density lipoproteins (LDL), and triglycerides with respect to age-matched controls (Zamorano et al., 1991; Adelekan et al., 2012; Buonuomo et al., 2019). Less favorable lipid profile in DS would be responsible for their increased risk of developing cerebrovascular events (Sobey et al., 2015; Buonuomo et al., 2019) or overweight/obesity (van Gameren-Oosterom et al., 2012; Buonuomo et al., 2019). Whether these alterations are due to a specific overexpressed gene on HSA21 is not known yet.

In the brain, a lipidomic study performed by Yu et al. (2018) reported altered lipid profile in prefrontal cortex samples collected from five DS (>60 years) individuals with respect to matched controls. Of 542 identified lipids, around 350 were reduced while the others were increased in DS frontal cortex (Yu et al., 2018). In particular, reduced levels of glycerophosphoethanolamines along with reduced glycerophospholipid metabolism were observed in DS (Yu et al., 2018). Furthermore, the ratio of cholesterol to phospholipid concentration, phosphatidylcholine, and phosphatidylethanolamine levels were all reduced in DS frontal cortex (Yu et al., 2018). These alterations were suggested to result from an impaired endocannabinoid signaling pathway in DS (Yu et al., 2018). In addition, in a very recent study, Hwang et al. (2019) demonstrated that DS fibroblast were characterized by reduced levels of sphingosine derivatives called long chain bases (LCBs), which seem to be responsible for nuclear membrane alterations associated with accelerated aging process in DS. Lower LCBs result from increased conversion to ceramide due to the activity of ceramide synthase (Hwang et al., 2019).

As a whole, the picture that emerges from these studies unravels a pathological metabolic phenotype of DS contributed by a number of different genes. Within this scenario, defects of mitochondrial function contribute to a general loss of cellular functions, most of which strictly depend on ATP availability (Coskun and Busciglio, 2012; Butterfield et al., 2014b; Valenti et al., 2018).

In catabolic tissues, mitochondria are the key organelles responsible for energy production that sustains a plethora of intracellular functions, which as a whole approximately consume about 90% of oxygen to generate ATP through oxidative phosphorylation (OXPHOS) (Rolfe and Brown, 1997). Mitochondria also participate in the oxidation, by ß-oxidation and Krebs cycle, of major macromolecules into key intermediates (metabolites) including pyruvate, fatty acids, and amino acids, which are sources of reducing equivalents, NADH, and/or FADH_2_.

A growing number of studies also demonstrated that loss of mitochondrial structure and function, which is associated with increased ROS production, contributes to DS pathological phenotypes (Valenti et al., 2018). This is not only the case for DS, but also other neurodevelopmental disorders such as Rett’s syndrome and autism (Valenti et al., 2014), as well as neurodegenerative diseases including Alzheimer’s disease and Parkinson’s disease (Johri and Beal, 2012).

Reduced rate of energy metabolism due to mitochondrial dysfunction significantly impairs neuronal functions, as well as neuronal development and survival (Mattson et al., 2008). Indeed, ATP production and redox homeostasis in brain mitochondria are essential to sustain neural developmental processes including cellular proliferation and differentiation, axonal and dendritic growth, and generation of synaptic spine and pre-synaptic compartments (Mattson et al., 2008). Mitochondrial deficits in DS is mainly the result of reduced efficiency to produce ATP through OXPHOS, together with decreased respiratory capacity and disruption of membrane potential and mitochondrial dynamics. These mitochondrial abnormalities have been observed in all DS cell types from peripheral to CNS cells, as reviewed in Valenti et al. (2018). Thus, mitochondrial dysfunction is considered an inherent feature of DS, associated with increased oxidative stress (Perluigi and Butterfield, 2012).

Vacca et al. analyzed mitochondrial function in fibroblasts and lymphoblastoid cells from DS subjects and found deficits in the OXPHOS system at multiple levels such as the complex I activity, the ATP synthase, the ADP/ATP translocator, and the adenylate kinase enzyme, ultimately leading to significant energy deficit and increased ROS production in mitochondria (Valenti et al., 2010, 2011). A severe bioenergetic deficit was also found in neural progenitor cells (NPCs) isolated from the hippocampus of Ts65Dn mice, a commonly used DS murine model, in which a deficit in cell proliferation was observed (Valenti et al., 2016). The reduced OXPHOS rate in DS could be associated with impairment of mitochondrial biogenesis, as observed in NPCs (Valenti et al., 2016), and in skin fibroblasts (Piccoli et al., 2013). Defects of mitochondrial biogenesis is the result of reduced mtDNA content as well as reduced levels of peroxisome proliferator-activated receptor gamma coactivator 1-alpha (PGC-1α), nuclear respiratory factor 1 (NRF-1), and mitochondrial transcription factor A (TFAM) (Valenti et al., 2016).

Several bioactive compounds display protective effects on mitochondrial signaling pathways, mitochondrial biogenesis, and respiration (Valenti et al., 2018). Both natural and synthetic compounds show the ability to dampen mitochondrial deficit and ROS overload in DS, and are likely to be promising therapeutic strategies to ameliorate DS pathological phenotypes (Valenti et al., 2018). Among possible candidates, recent studies show the protective effect of epigallocatechin gallate (EGCG), the major catechin in green tea, to induce neuronal plasticity (Martinez Cue and Dierssen, 2020) and mitochondrial function (Valenti et al., 2016), leading to cognitive rehabilitation in young adult DS (De La Torre et al., 2016).

## Peripheral and Brain Alterations in DS: a Look Into the Metabolic Defects Associated With Dysfunctions of Insulin Signaling Pathway

Among the metabolic alterations observed in DS subjects, dysfunctions of insulin signaling and related pathways are of interest. Although insulin or proteins belonging to the insulin signaling pathway are not encoded by genes located on HSA21, insulin signaling is at the crossroad among a number of intracellular events driving cell metabolism in terms of glucose, fatty acids, and proteins synthesis/utilization (Haeusler et al., 2018).

The frequency of metabolic diseases associated with defects of insulin and insulin-related pathways develop with high frequency in DS subjects. Furthermore, over the past few years, it has become clear that insulin also has profound effects in the central nervous system, where it regulates key processes such as energy homeostasis and neuronal functions (Arnold et al., 2018).

In the next sections, we summarize the accumulating evidence on peripheral and brain defects of insulin signaling and related pathway in DS. Current findings along with pathogenic factors and consequence in terms of disturbances are discussed.

## Peripheral Metabolic Alterations in DS and Possible Genetic Links

Obesity and subsequent metabolic disorders show higher prevalence in adult population with DS compared to individuals without DS. Among the weight-related and metabolic disorders present in DS individuals, glycemic dysregulation presents involving impaired fasting glucose, diabetes mellitus, dyslipidemia, and metabolic syndrome. DS individuals have higher body mass index (BMI) and body fat percentage (%BF) compared to age- and sex-matched persons without DS (Gonzalez-Aguero et al., 2011; Loveday et al., 2012; Gutierrez-Hervas et al., 2020). Interestingly, sex dimorphism appears in the DS population, being the BMI, %BF, and the proportion that is overweight is higher in females (Bell and Bhate, 1992; Slevin et al., 2014). The prevalence of childhood-onset autoimmune diabetes in the DS population is more than fourfold that of the general population. This increased prevalence of type 1 diabetes (T1D) could arise from the trisomy of genes on chromosome 21 (Bergholdt et al., 2006; Johnson et al., 2019). Type 2 diabetes mellitus (T2DM) occurs at an increased frequency at a relatively early age in DS subjects (Alexander et al., 2016). Furthermore, DS subjects show an increased rate of non-alcoholic fatty liver disease (NAFLD), which is closely associated with insulin resistance (Williams et al., 2011; Loomba et al., 2012).

Among the hormonal regulators of fat accumulation and energy balance, leptin levels in plasma correlate with adiposity in the general population but also in DS children and adolescents (Magni et al., 2004; Yahia et al., 2012). DS fetuses (Radunovic et al., 2003), adolescents (Gutierrez-Hervas et al., 2020), and adults (Proto et al., 2007) show lower leptin levels as compared to matched controls without DS. However, others have found that DS individuals had higher leptin levels compared to unaffected siblings (Proto et al., 2007; Magge et al., 2008). Inherent genetic basis for increased leptin resistance could explain the cases of hyperleptinemia not accompanied by hyperinsulinemia in individuals with DS (Tenneti et al., 2017).

**FIGURE 1:**
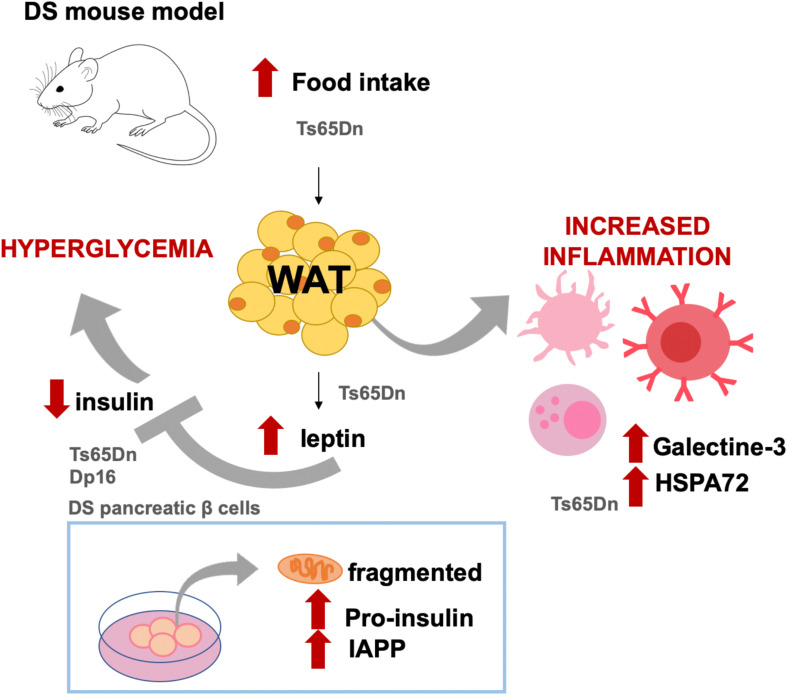
Diabetes-like phenotypes described in DS mouse models and cell cultures. Ts65Dn mice show increased food intake and body fat as compared to WT littermates. In this DS model, as compared to WT mice, leptin levels are also increased along with increased levels of molecules related to immune activation, such as galectine-3 and HSPA72. Leptin is an inhibitor of insulin, and in Ts65Dn (but also in Dp16 mice and in pancreatic islets from DS fetuses), insulin levels in plasma or secreted insulin are lower as compared to non-DS conditions. Both Ts65Dn and Dp16 models presented high plasmatic glucose levels, which has been mechanistically attributed to the triplication of RCAN1. In cell cultures, it has been shown that fetal T21 islets have fragmented mitochondria and present abnormal intracellular accumulation of pro-insulin and islet amyloid polypeptide (IAPP). The results illustrated came from Helguera et al. (2013), Peiris et al. (2016), and Fructuoso et al. (2018).

However, an inherent difficulty of human studies when trying to extract conclusions of metabolic features in different cohorts is the existence of confounding factors such a lifestyle and regional nutrition habits and preferences. Animal models provide a suitable alternative to investigate the impact of the HSA21 genes on the metabolic alterations in DS controlling for environmental factors. Recently, we investigated the effect of the trisomy in the metabolic-inflammatory axis and their relation with energy expenditure, energy intake, and fat accumulation in Ts65Dn mice, a partial trisomy DS model (Fructuoso et al., 2018; Figure 1). Ts65Dn mice presented increased fat mass and energy intake, along with increased leptin levels compared to WT (Fructuoso et al., 2018; Figure 1). Even so, Ts65Dn mice consumed more calories, suggesting that leptin would be ineffective in controlling the satiety. As compared to WT, Ts65Dn mice present lower levels of ghrelin in plasma, which in the general population has been associated with obesity (Buss et al., 2014). Ts65Dn mice also show increased glucose-stimulated response of the adipokine resistin and increase of plasma galectin-3 and HSP72 (Fructuoso et al., 2018; Figure 1), which are associated with autoimmunity (Krause et al., 2014; De Oliveira et al., 2015). Recently, higher levels of C-reactive protein (CRP), C3, and C4 complement factors have been reported in DS adolescents (Gutierrez-Hervas et al., 2020). Overall, the existing data support the idea of DS as a case of an impaired metabolic-inflammatory axis.

There is also a higher prevalence of diabetes in DS individuals compared to the general population (Anwar et al., 1998; Bergholdt et al., 2006). Fetal human pancreatic cells from DS fetuses show β-cell mitochondrial dysfunction, low ATP levels, and drastic decreased of their basal insulin secretion in comparison with cells from general population (Helguera et al., 2013). Polymorphisms of class II HLA genes located in chromosome 6 seem to be the major genetic factor of T1D in the general population (Jacobi et al., 2020). Interestingly, increased frequency of usual major TD1 risk allele of class II HLA has been described in DS individuals (Bergholdt et al., 2006). Studies that are more recent confirm that permanent neonatal diabetes in DS could have an autoimmune origin but are not HLA-associated (Johnson et al., 2019). Several known proteins encoded by HSA21 such as BACE2 (Esterhazy et al., 2011), RCAN1 (Peiris et al., 2016; Seo et al., 2019), and DYRK1A (Rachdi et al., 2014a), are expressed in peripheral organs and have been shown to be related to diabetes phenotypes. Therefore, besides the HLA region of chromosome 6, chromosome 21 may contain also candidate genes which overdose and may contribute to the metabolic disruptions, thus conferring increased TD1 risk in DS.

Supporting this idea, it has been shown that hyperglycemia is presented in Ts65Dn and Dp16 DS mouse models but not in Tc1 and Ts1Rhr mice (Peiris et al., 2016; Fructuoso et al., 2018), allowing the dissection of the region of chromosome 21 containing genes related to hyperglycemia (Figure 1). More specifically, this phenotype has been attributed to *RCAN1*, since transgenic mice overexpressing the human RCAN1-1 isoform present age-dependent hyperglycemia, impaired glucose tolerance, hypoinsulinemia, reduced β-cell secretion, reduced β-cell number, decreased insulin granule filling, and reduced mitochondria size in β-cell along with an aberrant mitochondrial reactive oxygen species production (Peiris et al., 2012). Moreover, overexpression of *RCAN* in mouse islets induced β-cell mitochondrial dysfunction and reduced ATP production, which may cause the inhibition of insulin secretion (Peiris et al., 2016).

Recently, it has been shown that a single extra copy of *RCAN1* in the mouse could also cause insulin resistance and pyruvate intolerance, probably though GSK3β or NIK regulation by increasing hepatic glucose production and expression of gluconeogenic genes (Seo et al., 2019). Impaired insulin secretion has also been attributed to the overexpression of another HSA21 gene, *BACE2*. *BACE2* gain-of-function experiments in INS1E cell cultures decreased cell proliferation and insulin secretion, along with increased mitochondrial metabolism and ROS levels (Alcarraz-Vizan et al., 2015). Conversely, loss of function experiments improved insulin secretory response in rat pancreatic β-cells (Alcarraz-Vizan et al., 2015).

Pharmacological inhibition of DYRK1A in both mice and human cells leads to proliferation of β-cell (Wang et al., 2015; Belgardt and Lammert, 2016) and improves glycemic control in mice (Liu et al., 2020). *Dyrk1A* is highly expressed and induces the expression and nuclear accumulation of p27Kip1 in the adipose tissue and pancreas (Rachdi et al., 2014b). Mice overexpressing *Dyrk1A* showed increased β-cell mass and improved glucose homeostasis (Rachdi et al., 2014a), probably mediated by p27Kip1 since it acts as a negative regulator of proliferation via the inhibition of cyclin-CDK activity (Lloyd et al., 1999). Moreover, mice overexpressing *Dyrk1A* are characterized by reduced fat mass, increased Thr(P) (356)-GSK3β in the white adipose tissue, and downregulation of adipogenic proteins (Song et al., 2015). This could be explained in part because DYRK1A specifically inhibits GSK3β (Song et al., 2015), a transcription factor associated with adiposity and obesity (Pearce et al., 2004). Accordingly, *Dyrk1A* haploinsufficiency in mice produces severe glucose intolerance, reduced β-cell mass, and decreased β-cell proliferation (Rachdi et al., 2014b).

The existing data suggest that the sole overexpression of DYRK1A does not reproduce peripheral phenotypes associated to obesity, such as fat accumulation and insulin deficiency, in DS humans. However, only *Dyrk1A* overexpression might be relevant for feeding behavior and energy balance, since transgenic mice exhibit increased food intake (Hong et al., 2012). Hypothalamic nuclei expressed the neuropeptide Y (NPY), an orexigenic neuropeptide that promotes food intake (Spiegelman and Flier, 2001). *Dyrk1A* expression can be modulated by NPY through the PKA-CREB up-stream pathway, which in turn activates a positive feedback loop where Sir2-dFOXO induces NPY gene expression (Hong et al., 2012). In fact, the hypothalamus of *Dyrk1A-*overexpressing mice display reduced FOXO acetylation and increased NPY expression (Hong et al., 2012). Considering that *Dyrk1A* is triplicated in DS, the reinforced positive NPY feedback mediated by *Dyrk1A* could contribute to the increased energy intake observed DS models and obesity in DS (Fructuoso et al., 2018).

Other HSA21 encoded proteins, such as ADAMTS1, APP, GABPA, HSPA13, LIPI, NRIP1, and hsa-mir-99a, have also been associated with obesity. Specifically, in Genome Wide Association Studies (GWAS), Kunej and collaborators describe in the general population a genetic association between quantitative traits loci (QTL) for body weight (BW276_H) and these seven loci located in the HSA21 (Kunej et al., 2013).

Certainly, besides the overexpression of coding and non-coding regions of the HSA21, genes located in other chromosomes whose expression is dysregulated could also take part in the DS phenotypes. This genome-wide transcriptional deregulation could be due to epigenetic modifications. In trisomy 21 discordant twins as well as the Ts65Dn/WT mouse cells, the chromatin modifications produced by nuclear compartments of T21 cells affect the overall (Letourneau et al., 2014; Antonarakis, 2017). Alternatively, it could also be a result of the sole presence of extra DNA material in the nucleus that may favor specific gene-regulatory programs independently of the sequence (Letourneau et al., 2014). In fact, chromosomal contacts maps allow the inference of loci associated with BMI as shown for reciprocal duplication carriers of the distal 16p11.2 that predisposes to a highly penetrant form of obesity (Loviglio et al., 2017). More recently, Espeso and coworkers showed inter- and intra-chromosomal contacts linking SCZ and BMI risk sequences (Espeso-Gil et al., 2020). Those present massive enrichment for brain-specific expression quantitative trait loci related to adipogenesis and lipid regulation, dopaminergic neurogenesis and neuronal connectivity, and reward- and addiction-related pathways (Espeso-Gil et al., 2020).

Finally, besides obesity, DS populations show the endocrine condition of hypothyroidism, mainly as subclinical hypofunction (Graber et al., 2012; Lavigne et al., 2017). This endocrine abnormality may contribute to the increased fat accumulation in DS people because the activation of gene pathways controlling thermogenesis, glucose homeostasis, and fat oxidation can be modulated by thyroid hormones (Mullur et al., 2014). Interestingly, medullary thymocytes from DS patients show altered the mRNA levels of the autoimmune regulator (AIRE) gene, sited on HSA21, resulting in the consequent deregulated expression of *INSULIN* and *CHRNA1*genes (Gimenez-Barcons et al., 2014). *Dyrk1A* could affect early thyroid morphogenesis through the up-regulation of transcription factors (*Nkx2–1*, *Foxe1*, and *Pax8*) relevant for this embryonic process. Consequently, *Dyrk1A*-overexpressing mice show significantly thicker but less functional thyroids, as shown by the lower T4 hormone excretion and the tendency to have increased plasma TSH (Kariyawasam et al., 2015).

## Brain Insulin Resistance: a Key Metabolic Alteration in Aging and Neurodegeneration

Brain represents only ∼2% of body weight but has a high metabolic demand compared to other tissues. Among brain cells, neurons require a lot of energy to support signaling (Magistretti and Allaman, 2018) and most of this energy is produced through glucose oxidative metabolism (Magistretti and Allaman, 2018). While glucose uptake and metabolism are finely regulated by insulin in peripheral tissues/organs, the brain was thought to be not affected by insulin in terms of glucose uptake (Arnold et al., 2018; Nakabeppu, 2019).

Recent advances in the comprehension of brain functions highlighted that the actions of insulin are more pronounced in the central nervous system than previously thought. Insulin plays a major role in the regulation of gene expression and cellular metabolism, both events that sustain neuronal activity and synaptic plasticity mechanisms (Arnold et al., 2018). Alterations of brain insulin signaling have been associated with a higher risk of developing age-related cognitive decline and neurodegenerative diseases (Butterfield et al., 2014a; Arnold et al., 2018). In particular, among altered processes identified to precede the appearance of frank symptoms and neuropathology in AD, brain insulin resistance greatly contributes to the long preclinical period during which often only subtle symptoms are evident (Stanley et al., 2016).

Insulin resistance is generally defined as an insufficient response to insulin by target cells (Konner and Bruning, 2012; Butterfield et al., 2014a) and represents a central feature of metabolic disorders, including T2DM and obesity (Lazar, 2005; Kahn et al., 2006; Bluher, 2016; Lin et al., 2016). This lack of response might be due to downregulation of insulin receptors (IR) or defective activation of the insulin signaling cascade. All the research-based evidence collected so far suggests that the molecular mechanisms responsible for the development of systemic and brain insulin resistance are quite similar (Butterfield et al., 2014a; Verdile et al., 2015). Indeed, a significant overlap in risk, comorbidity, and pathophysiological mechanisms exists across AD and T2DM or obesity (Butterfield et al., 2014a; Verdile et al., 2015; Arnold et al., 2018). For that reason, AD was defined as “diabetes of the brain,” or type 3 diabetes (Arnold et al., 2018; De La Monte, 2019).

Thus, insulin resistance is central to our understanding of shared features between AD and metabolic disorders. It was reported that more than hyperglycemia (which is a peculiar condition of diabetes) and associated effects, the insulin resistance phenomenon by itself plays a crucial role during the preclinical stage of AD (Morris et al., 2014, 2016; Arnold et al., 2018). This is mainly due to the role of insulin in regulating brain functions (Arnold et al., 2018). In the brains of healthy individuals, insulin signaling activation has been shown to be involved in several pathways including: (1) improvement of neurite outgrowth, (2) releasing and uptake of catecholamine, (3) regulation of trafficking of ligand-gated ion channels, (4) expression and localization of GABA, N-methyl-D-aspartate (NMDA) and α-amino-3-hydroxy-5-methyl-4-isoxazole propionic acid (AMPA) receptors, and (5) modulation of synaptic plasticity [long-term potentiation (LTP) and long-term depression (LTD)]. Furthermore, insulin plays a role in the development and maintenance of excitatory synapses and promotes dendritic spine formation and excitatory synapse development. Finally, by inhibiting apoptosis, insulin promotes neuronal survival. Importantly, development of brain insulin resistance impairs all these functions (Arnold et al., 2018).

**FIGURE 2:**
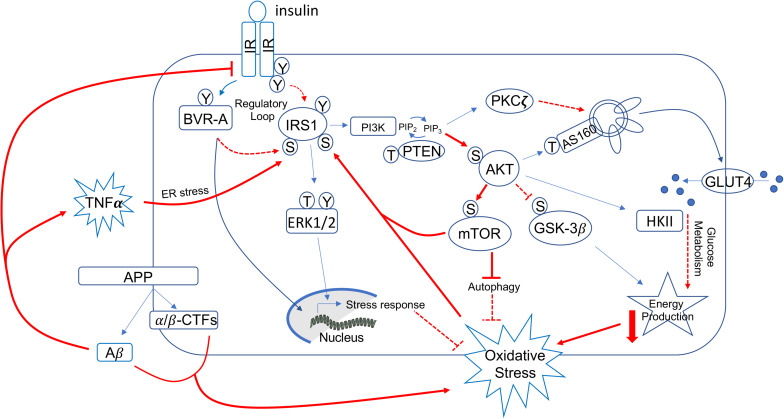
Schematic representation of insulin signaling with highlighted in red pathways found to promote brain insulin resistance in AD and DS. Under physiological conditions, the activation of insulin signaling requires the binding of insulin to the insulin receptor (IR), which auto-phosphorylates on Tyr residues (e.g., Tyr1158/1162/1163) and promotes the receptor tyrosine kinase-mediated phosphorylation of its substrate (IRS1) on specific Tyr residues (e.g., 632). In parallel, IR phosphorylates BVR-A on specific Tyr residues and activates BVR-A to function as Ser/Thr/Tyr kinase. Then, as part of a regulatory loop, BVR-A phosphorylates IRS1 on inhibitory Ser residues (Ser307/312/616) to avoid IRS1 aberrant activation in response to IR. Once activated, IRS1 works as a scaffold protein, driving the activation of the two main harms of the insulin signaling: (1) the MAPK pathway (ERK1/2) mainly involved in gene transcription and (2) the PI3K/Akt axis that is critical for linking upstream effectors (IR and IRS1) with downstream proteins mediating insulin neurotrophic outcomes. Activation of the PI3K/Akt axis is regulated by the phosphatase PTEN, which reduces PIP3 levels required for Akt activation as well as for increasing the expression of PKCζ. Akt promotes the phosphorylation of several targets, among which are: (1) GSK3β (on Ser9, inhibitory site), which has a role energy production; (2) mTOR (on Ser2448, activating site), which regulates protein synthesis and autophagy; and (3) AS160 (on Thr642, activating site). This latter, together with PKCζ, is responsible for the translocation of GLUT4-containing vesicles to the plasma membrane to mediate glucose uptake. Furthermore, Akt stimulates the upregulation of HKII, which is a pivotal enzyme involved in glucose metabolism and thus energy production. During the development of brain insulin resistance, a dysregulation of a number of these proteins was observed. In particular, brain insulin resistance phenomenon is characterized by key events such as reduced IR protein levels and/or increased IRS1 inhibitory phosphorylation levels (e.g., Ser307, Ser636), that are responsible for the uncoupling between IR and IRS1. As result, despite insulin binding to IR, IR-mediated activation of IRS1 does not occur. Downstream from IRS1, the aberrant activation of the PI3K/Akt/mTOR axis was observed. This event promotes the uncontrolled activation of mTOR able to phosphorylate IRS1 on inhibitory sites. Moreover, brain insulin resistance was associated with increased Aβ production, which was in turn responsible for IR internalization and thus reduced IR protein available at the plasma membrane to bind insulin. Furthermore, increased inflammatory processes promote a rise of TNFα levels, which favors the activation of stress-induced kinases (i.e., JNK, IKK, PKR) and ER stress, which are all events known to favor IRS1 inhibition. Finally, many of the above described pathways are associated with increased oxidative stress levels, which further contribute to IRS1 inhibition and thus insulin signaling deregulation. Red plain lines/arrows: increased during AD and DS; red dotted lines/arrows: reduced during AD and DS.

Mechanisms identified to prompt the development of brain insulin resistance (Figure 2) include, among the others: reduced insulin transport across the blood brain barrier (BBB) (Craft et al., 1998; Sartorius et al., 2015; Rhea et al., 2020), uncontrolled activation of inflammatory processes (Bomfim et al., 2012; Lourenco et al., 2013), increased Aβ oligomers (Lourenco et al., 2013; Seixas Da Silva et al., 2017), increased oxidative stress levels (Butterfield et al., 2014a; Barone et al., 2016; Maciejczyk et al., 2019; Sharma et al., 2019), aberrant activation of the mammalian target of rapamycin (mTOR) (Carlson et al., 2004; Caccamo et al., 2010, 2011; Bove et al., 2011; Copps and White, 2012; Oddo, 2012; Perluigi et al., 2014; Tramutola et al., 2015; Barone et al., 2016), impaired function of biliverdin reductase-A (BVR-A) (Barone et al., 2016, 2019; Triani et al., 2018; Sharma et al., 2019), and fatty acids dysmetabolism (Spinelli et al., 2017; Banks et al., 2018; Melo et al., 2020).

From a molecular point of view, the activation of insulin signaling requires the binding of insulin to IR, which auto-phosphorylates and promotes the phosphorylation of the insulin receptor substrate 1 (IRS1) on specific Tyr residues (e.g., 612 and 632). Once activated, IRS1 works as scaffold protein driving the activation of the two main arms of the insulin signaling pathway: (1) the phosphatidylinositol 3-kinase/3-phosphoinositide-dependent protein kinase 1/protein kinase B (PI3K/PDK1/Akt) and (2) the mitogen-activated protein kinase (MAPK) pathways (White, 2003; Figure 2). Instead, development of insulin resistance (both in the brain and peripheral tissues) occurs following the inhibitory phosphorylation of IRS1 on specific serine residues (307, 312, 636), which preclude IRS1 to interact with IR. As result, cells become insensitive to the action of insulin since the pathway cannot be activated downstream from IR/IRS1 axis (Figure 2).

Studies performed in human and mouse models of AD highlighted the role of inflammation and in particular the inflammatory cytokine tumor necrosis factor alpha (TNF-α), which leads to the activation of specific kinases [i.e., Jun N-terminal kinase (JNK), I kappa B kinase (IKK), and protein kinase R (PKR)] (Bomfim et al., 2012; Lourenco et al., 2013) and endoplasmic reticulum (ER) stress (PKR-mediated phosphorylation of eIF2α) (Lourenco et al., 2013) responsible for IRS1 inhibition (Figure 2). Elevated TNF-α seems to be a consequence of the brain accumulation and the impact of Aβ oligomers, which were demonstrated to induce brain IRS1 inhibition through a mechanism involving either reduced IR exposure at the plasma membrane (Zhao et al., 2008; De Felice et al., 2009; Forny-Germano et al., 2014) or increased ER stress via PKR (Lourenco et al., 2013) in AD (Figure 2). These molecular events promote synapses loss and memory impairment (De Felice et al., 2009; Talbot et al., 2012; Barone et al., 2019). Rise of inflammation seems to be a condition sufficient but not necessary during the development of IR, since disruption of inflammatory pathways, e.g., by JNK deletion, did not attenuate the pathological features of the early stage of peripheral insulin resistance (Lee et al., 2011).

This aspect is quite fascinating because it means that other mechanisms could be responsible for the early alterations of insulin signaling pathway. In particular, our group highlighted the role of BVR-A as regulator of insulin signaling (Barone et al., 2011a, b, 2016, 2019; Di Domenico et al., 2013b; Triani et al., 2018; Cimini et al., 2019; Sharma et al., 2019; Figure 2). Impairment of BVR-A and accumulation of markers of insulin resistance were observed in AD subjects (Barone et al., 2011a, b; Di Domenico et al., 2012). Loss of BVR-A leads to the hyper-activation of IRS1 without a concomitant activation of Akt downstream from IRS1 in animal model of AD (Triani et al., 2018; Sharma et al., 2019) as well as *in vitro* (Miralem et al., 2016; Sharma et al., 2019), suggesting an overall uncoupling among the proteins responsible for insulin action within the cells. In addition, these alterations were associated with increased Tau phosphorylation and Aβ production (Triani et al., 2018; Sharma et al., 2019). Intriguingly, the ability to overcome brain insulin resistance requires sufficient expression of key proteins, including BVR-A (Barone et al., 2019).

Furthermore, several studies report about the overactivation of the PI3K/Akt/mTOR pathway that occurs during the early phases of AD (Pei et al., 2008; Caccamo et al., 2010; Tramutola et al., 2015), leading to impaired glucose metabolism, defects in energy production, and aberrant regulation of protein synthesis and degradation (Oddo, 2012; Tramutola et al., 2015; Di Domenico et al., 2017). In AD, brain insulin resistance is sustained by overactivation of the PI3K/Akt/mTOR axis, which inhibits IRS1 through a negative feedback mechanism (Tramutola et al., 2015; Figure 2). Aberrant mTOR activation is associated with increased AD neuropathological markers both in humans and animal models of the pathology (Caccamo et al., 2010, 2011; Orr et al., 2014; Tramutola et al., 2015; Barone et al., 2016, 2019; Triani et al., 2018). In addition, chronic mTOR overactivation has been linked to increased inflammatory process (Liu et al., 2016; Paschoal et al., 2017) and oxidative stress levels (Tramutola et al., 2016; Figure 2). Interestingly, rapamycin (a well-known mTOR inhibitor) (Caccamo et al., 2010; Spilman et al., 2010) mitigates AD pathology and cognitive dysfunctions in mice fed with an insulin resistance-inducing diet (Orr et al., 2014).

Notwithstanding metabolic disorders that might accelerate the risk to develop brain insulin resistance, recent studies highlight that brain insulin resistance can occur independently from peripheral alterations both during aging and in AD. Decreased insulin levels and reduced binding of insulin to insulin receptors were reported in the cortex of elderly individuals without dementia (Frolich et al., 1998).

Furthermore, reduced insulin signaling activation was observed in the brain of AD subjects without T2DM (Rivera et al., 2005; Steen et al., 2005; Moloney et al., 2010; Talbot et al., 2012; Tramutola et al., 2015). In two independent cohorts of post-mortem brains samples collected from AD or mild cognitive impairment (MCI) subjects, consistent defects in the basal levels and/or activation of proteins belonging to insulin signaling were described (Talbot et al., 2012). These alterations were positively associated Aβ and tau levels while they were negatively correlated with scores of cognitive and memory tasks (Talbot et al., 2012). Interestingly, the associations remained significant even after controlling for Aβ and Tau lesions, suggesting that brain insulin resistance contributed independently to the observed cognitive impairment (Talbot et al., 2012).

## Brain Insulin Resistance Is a Feature of Down Syndrome

DS brains share many common neuropathological features with AD, including brain insulin resistance (Tramutola et al., 2020). Interestingly, our group highlighted for the first time that markers of brain insulin resistance are evident in DS brains even before the development of AD pathology (Tramutola et al., 2020), suggesting that these alterations might support the mechanisms associated with intellectual disability as well as the early onset of AD in people with DS (Lott and Head, 2019).

Causes for brain insulin resistance development in DS could be various, considering that DS phenotype shows both peripheral and brain alterations that would trigger the impairment of the brain insulin signaling pathway. The peripheral alterations presented above in this review suggest that DS subjects might be potentially more susceptible to developing brain insulin resistance than other children. Indeed, growing evidence highlight the role of metabolic defects as a risk factor for cognitive impairments also in DS (Caracausi et al., 2018; Head et al., 2018; Vacca et al., 2019).

With regard to brain alterations, accumulation of toxic catabolites (Caracausi et al., 2018; Gross et al., 2019) or dysfunction of key metabolic pathways were identified as crucial determinants triggering neuronal dyshomeostasis and neurodegeneration in DS (Head et al., 2018; Lott and Head, 2019; Vacca et al., 2019).

Interestingly, DS brains present all the alterations described in the previous section that are known to be associated with the development of brain insulin resistance. DS brain is characterized by significant increased Aβ oligomers production and Aβ accumulation (Nistor et al., 2007; Cenini et al., 2012; Lott and Head, 2019) along with a pro-inflammatory state (Nistor et al., 2007; Wilcock et al., 2015; Lott and Head, 2019). Inflammation occurs early in life (Wierzba-Bobrowicz et al., 1999) with increased glial activation (Wilcock, 2012; Wilcock et al., 2015) and prominent levels of pro-inflammatory cytokines in serum from both adults (Convertini et al., 2016; Iulita et al., 2016) and children (Zhang et al., 2017). These neurotoxic events are associated with defect of autophagy process (Ahmed et al., 2013; Perluigi et al., 2014; Colacurcio et al., 2018; Bordi et al., 2019). In that regard, we reported about the hyperactivation of the PI3K/Akt axis along with aberrant mTOR activation in the frontal cortex of both DS and DS individuals who develop AD pathology (DSAD) with respect to age-matched controls (Perluigi et al., 2014). Moreover, increased oxidative stress levels leading to protein and lipid oxidative damage accumulation were observed (Perluigi et al., 2011; Cenini et al., 2012; Di Domenico et al., 2013a, 2014; Tramutola et al., 2016; Barone et al., 2018). In addition, impairment of BVR-A was found in both human and mouse brain (Di Domenico et al., 2015). Last but not least, induction of ER stress with increased PKR activation and eIF2 phosphorylation of have been observed in humans and Ts65Dn mouse model of DS (Lanzillotta et al., 2018; Zhu et al., 2019).

**FIGURE 3:**
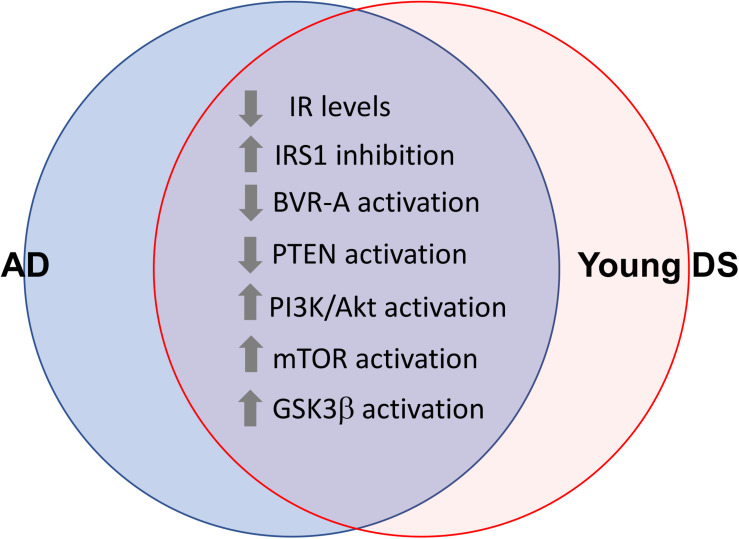
Shared alterations between young DS and AD brain. Proteins of the insulin signaling pathway found to be dysregulated both in young DS (<40 years) and AD brain. These alterations occur quite early and even before the development of AD pathology in DS brain, thus representing a risk factor for AD development in DS. With regards to GSK3β the loss of Akt-mediated inhibition was observed (Tramutola et al., 2020). Considering that GSK3β is a constitutively activated kinase, reduced inhibitory processes might favor its aberrant activation in young DS.

These lines of evidence strongly suggest that brain insulin resistance can develop also in DS. Indeed, in a recent paper, Tramutola et al. (2020) reported about reduced IR protein levels and increased IRS1 inhibition in DS brains, even before the development of AD pathology. From a molecular point of view, reduced sensitivity to the effects of insulin in DS brains is associated with an overall impairment of the insulin signaling pathway mainly characterized by: (1) reduced phosphatase and tensin homolog (PTEN) activation, which drives the observed increased Akt activation in young DS; (2) loss of Akt-mediated inhibition of glycogen synthase kinase-3 beta (GSK3β), which results in more active GSK3β in young DS; (3) reduced protein kinase C zeta (PKCζ) protein levels, which account for reduced translocation of GLUT4 to the plasma membrane and thus reduced insulin-mediated glucose uptake in the brain; and (4) reduced glucose metabolism due to reduced hexokinase II protein levels as well as reduced mitochondrial complexes levels in young DS (Tramutola et al., 2020; Figure 3). From a pathological point of view, these alterations were associated with increased APP cleavage products and elevation of TNFα levels (Tramutola et al., 2020).

In light of the role of the insulin signaling pathway in regulating synaptic plasticity and cognitive functions, alterations of proteins belonging to the insulin signaling but not APP cleavage products or TNFα levels were significantly associated with reduced synaptic proteins levels, i.e., syntaxin and postsynaptic density 95 (PSD95), in DS with respect to age-matched control (Tramutola et al., 2020). Of note, reduced levels of syntaxin and PSD95 proteins also were associated with reduced mitochondrial complexes levels in DS (Tramutola et al., 2020), thus highlighting the tight link existing among defects of insulin signaling pathway, mitochondrial alterations, and mechanisms regulating synaptic plasticity in DS.

As reported by our group, intranasal rapamycin (a well-known mTOR inhibitor; Guertin and Sabatini, 2009) administration to Ts65Dn mice promoted neuroprotective effects including reduced AD pathological hallmarks (Aβ and Tau levels), reduced oxidative stress levels, and increased synaptic proteins levels and amelioration of cognitive functions (Tramutola et al., 2018; Di Domenico et al., 2019). Interestingly, rapamycin was effective in reducing IRS1 inhibition while downstream from IRS1-increased PTEN activation and promoted the normalization of the Akt/GSKβ axis activation in the hippocampus of Ts65Dn mice (Tramutola et al., 2018). Hence, these results further support the hypothesis that cognitive deficits in DS might be mediated by the dysfunction of insulin signaling in the brain. By taking in mind that mTOR is activated in response to insulin while hyperactive (aberrant) mTOR promotes IRS1 inhibition (Copps and White, 2012), observations collected with regard to the use of rapamycin spurs the necessity to better understand whether hyperactivation of mTOR results from the dysfunction of the insulin signaling pathway or is a primary cause of the observed impairment of the pathway in DS. Notwithstanding, it appears clear that all the observed defects are already evident in young DS individuals (Perluigi et al., 2014; Tramutola et al., 2020), thus representing a risk factor for the development of AD pathology with age. Actually, improving brain insulin signaling activation represents a promising therapeutic strategy to rescue cognitive functions in AD (Arnold et al., 2018; Batista et al., 2018; Barone et al., 2019; Zhang et al., 2020).

## Conclusion

DS subjects represent a unique population that needs to be carefully followed during the entire course of their life. One of the main defects caused by trisomy of HSA21 is metabolic defect. Indeed, DS individuals show a pattern of metabolic defects that contribute to increase the risk of developing chronic diseases such as diabetes or AD-like dementia. This is a crucial aspect considering that metabolic diseases characterized by insulin resistance, such as obesity and diabetes, are associated with a progressive cognitive decline as well as with a risk of developing dementia in the general population. Keeping this in mind, cognitive decline occurs in DS because their genetic background could be further aggravated by the onset of metabolic disorders, which could in turn foster the development of AD in DS patients. Despite knowledge concerning the molecular mechanisms potentially involved in the progression of peripheral and brain metabolic disorders in DS, early alterations are still unknown and require further investigations. Indeed, identifying these alterations during their initial stage could have the advantage of being caught early enough to stop/delay their progression. In this picture, defects of insulin signaling seem to occur early in life at both peripheral and brain levels and persist with aging in DS. Furthermore, at the cellular level, the dysfunction of insulin signaling pathway crosses with dysfunctions of proteins encoded by genes on HSA21 (e.g., *Dyrk1A*, *RCAN*, and *APP)*, thus contributing to worsening a pre-existing condition defined on a genetic background. This aspect is fascinating especially in light of the role of insulin signaling pathway in regulating energy metabolism.

While collected results about metabolic defects are uncovering novel aspects associated with DS condition, the field is quite challenging, particularly with regard to brain alterations. The genetic background makes DS a quite complex condition and it is unthinkable that a single treatment could be enough to rescue genetic-driven defects. However, what we can do is to try to improve brain alterations, particularly treating AD in DS or even preventing/delaying AD progression in DS. Indeed, to our knowledge, no therapeutic strategies are available, despite this representing a big issue even in light of the fact that life expectancy is increased in DS. Thus, future research should focus on the identification of altered mechanisms that can be improved through the use of available drugs. Brain insulin resistance could be one of these molecular pathways, and we have examples of successful strategies especially in AD. Intranasal insulin administration has been proved to ameliorate cognitive decline in AD subjects and the effects are even better when insulin is administered in the early stages of the pathology (e.g., MCI) (Craft et al., 2012, 2017; Claxton et al., 2013, 2015). Furthermore, the use of antidiabetic drugs including metformin and GLP1 mimetics showed promising results in animal models of AD (Jha et al., 2017; Holscher, 2018). Moreover, considering that insulin signaling activation regulates mitochondrial functions (Butterfield et al., 2014a; Abad et al., 2019; Wardelmann et al., 2019) and that mitochondria are dysfunctional in DS (Mollo et al., 2020), it is conceivable to think rescuing insulin signaling activation in DS would be beneficial also with respect to mitochondrial performances. Metformin, a well-known drugs used to treat insulin resistance, was effective in recovering mitochondrial structure and functions in trisomic cells (Izzo et al., 2018). Furthermore, several drugs including insulin, metformin, and incretin mimetics have shown to improve insulin resistance and mitochondrial functions *in vitro* and are currently under evaluation for their beneficial effects in neurodegenerative diseases and aging process in humans (reviewed in Jha et al., 2017; Holscher, 2018; Lee et al., 2018).

In conclusion, drawing the molecular signature underlying alterations of insulin signaling in DS is a key challenge to identifying novel drug targets and set-up new prevention strategies aimed to reduce metabolic disorders as well as their impact on cognitive decline in DS.

## Author Contributions

All authors conceived the work, took part to the scientific discussion, and wrote the manuscript.

## Conflict of Interest

The authors declare that the research was conducted in the absence of any commercial or financial relationships that could be construed as a potential conflict of interest.
